# Improved production of secreted heterologous enzyme in *Bacillus subtilis* strain MGB874 via modification of glutamate metabolism and growth conditions

**DOI:** 10.1186/1475-2859-12-18

**Published:** 2013-02-18

**Authors:** Kenji Manabe, Yasushi Kageyama, Takuya Morimoto, Eri Shimizu, Hiroki Takahashi, Shigehiko Kanaya, Katsutoshi Ara, Katsuya Ozaki, Naotake Ogasawara

**Affiliations:** 1 Biological Science Laboratories, Kao Corporation, 2606 Akabane, Ichikai, Haga, Tochigi 321-3497, Japan; 2 Graduate School of Biological Science, Nara Institute of Science and Technology, 8916-5 Takayama, Ikoma, Nara 630-0101, Japan; 3 Analytical Science Laboratories, Kao Corporation, 2606 Akabane, Ichikai, Haga, Tochigi 321-3497, Japan; 4 Medical Mycology Research Center, Chiba University, 1-8-1 Inohana, Chuo-ku, Chiba 260-8673, Japan; 5 Graduate School of Information Science, Nara Institute of Science and Technology, 8916-5 Takayama, Ikoma, Nara 630-0101, Japan; 6 Fundamental Technology Research Laboratories, Kao Corporation, 623 Zi Ri Rd, Minhang Dist, Shanghai 200241, China

**Keywords:** *Bacillus subtilis*, Protein secretion, Genome reduction, Glutamate metabolism

## Abstract

**Background:**

The *Bacillus subtilis* genome-reduced strain MGB874 exhibits enhanced production of exogenous extracellular enzymes under batch fermentation conditions. We predicted that deletion of the gene for RocG, a bi-functional protein that acts as a glutamate dehydrogenase and an indirect repressor of glutamate synthesis, would improve glutamate metabolism, leading to further increased enzyme production. However, deletion of *rocG* dramatically decreased production of the alkaline cellulase Egl-237 in strain MGB874 (strain 874∆rocG).

**Results:**

Transcriptome analysis and cultivation profiles suggest that this phenomenon is attributable to impaired secretion of alkaline cellulase Egl-237 and nitrogen starvation, caused by decreased external pH and ammonium depletion, respectively. With NH_3_-pH auxostat fermentation, production of alkaline cellulase Egl-237 in strain 874∆rocG was increased, exceeding that in the wild-type-background strain 168∆rocG. Notably, in strain 874∆rocG, high enzyme productivity was observed throughout cultivation, possibly due to enhancement of metabolic flux from 2-oxoglutarate to glutamate and generation of metabolic energy through activation of the tricarboxylic acid (TCA) cycle. The level of alkaline cellulase Egl-237 obtained corresponded to about 5.5 g l^-1^, the highest level reported so far.

**Conclusions:**

We found the highest levels of production of alkaline cellulase Egl-237 with the reduced-genome strain 874∆rocG and using the NH_3_-pH auxostat. Deletion of the glutamate dehydrogenase gene *rocG* enhanced enzyme production via a prolonged auxostat fermentation, possibly due to improved glutamate synthesis and enhanced generation of metabolism energy.

## Background

*Bacillus subtilis* is attractive for industrial use for a variety of reasons, including its rapid growth rate, ability to secrete proteins into the medium, and its ‘generally regarded as safe’ (GRAS) status [1,2]. *B. subtilis* is also one of the best-characterized model microorganisms, as a result of extensive biochemical, genetic, and molecular biological studies [3,4]. *B. subtilis* has been used for the industrial production of enzymes for detergents, foods, and beverages. In industrial-scale production of enzymes, improvement of production levels is a major topic of interest.

We previously reduced the size of the *B. subtilis* genome by deleting unnecessary regions in order to construct a simplified microbial cell ‘factory’ for recombinant enzyme production. To do this, we constructed a multiple-deletion mutant strain, MGB874, via the sequential deletion of 865 genes (874 kb; 20.7%) from *B. subtilis* strain 168 [5,6]. As compared to strain 168, strain MGB874 shows enhanced production of the exogenous secreted alkaline cellulase Egl-237 [7] and alkaline protease M-protease [8] from plasmid-encoded genes in modified 2xL-Mal medium, a model medium for industrial protein production.

**Figure 1:**
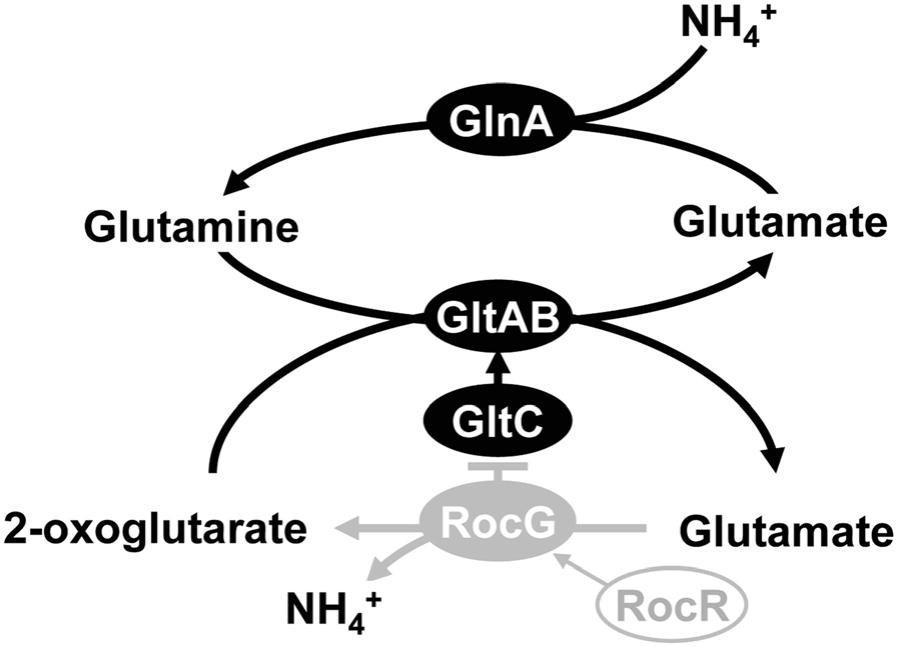
**Major reactions and regulation involved in glutamate metabolism in *B. subtilis.*** Proteins are shown as ovals. RocG, glutamate dehydrogenase; GltAB, glutamate synthase (GOGAT); GlnA, glutamine synthetase (GS). In *B. subtilis*, glutamate can be degraded by RocG. *B. subtilis* has a glutamine synthetase-glutamate synthase (GS-GOGAT) pathway for assimilation of ammonia. The RocR and GltC transcription factors positively regulate *rocG* and *gltAB*, respectively, and GltC can be inhibited via interaction with RocG. Open and closed gray ovals indicate proteins corresponding to genes that have been deleted or inactivated, respectively, in strain MGB874. Deletion of *rocR* in strain MGB874 decreases expression of *rocG*, which leads to an increase in expression of *gltAB* due to activation of GltC via disinhibition by RocG.

We have also shown that deletion of the *rocR* gene is an important contributor to the high level of enzyme production that we observe in genome-reduced strain MGB874 [9]. The RocR protein is a positive regulator of genes related to the arginine degradation pathway, including RocG, a major glutamate dehydrogenase [10-13]. RocG has another role as a regulatory protein that inhibits GltC, a transcription activator protein of the *gltAB* operon, which encodes glutamate synthase [14]. Thus, in strain MGB874, deletion of *rocR* not only inhibits glutamate degradation pathway but also activates the glutamate synthesis pathway (Figure 1). We proposed that this change of glutamate metabolism in strain MGB874 increases the flux from 2-oxoglutarate to glutamate, which might lead to increased syntheses of the other amino acids via transamination, finally resulting in enhanced enzyme production [9].

Additionally, we found that RocG also serves as an important factor influencing enzyme production by helping to prevent acidification of the growth medium. Decreased expression of *rocG* reduces the level of deamination of glutamate, a major cellular ammonia-releasing reaction [15], and leads to a decrease in the external pH during strain MGB874 cultivation [16]. We found that the decreased external pH impaired production of the alkaline α-amylase AmyK38, accompanied by the induction of expression of *htrA* and *htrB*, which encode serine-type surface proteases and are known to be CssRS dependent [16]. In *B. subtilis*, the CssRS two-component system responds to the accumulation of misfolded proteins at the membrane-cell wall interface [17]. Alkaline α-amylase AmyK38 is thought to fold ineffectively at acidic external pH, leading to secretion stress. Therefore, at least in terms of the production of the alkaline α-amylase AmyK38, RocG appears to have a positive role in preventing acidification of the growth medium.

The aim of the present study was to enhance enzyme production in genome-reduced strain MGB874 through further optimization of glutamate metabolism. Belitsky *et al*. reported that *rocG* is still expressed at a low level due to read-through transcription of the upstream gene *yweA*, even in the absence of the RocR activator sequence [18]. Thus, deletion of *rocG* might release repression of *gltAB* in strain MGB874 completely, further enhancing enzyme production. However, we previously observed that deletion of *rocG* in strain MGB874 (strain 874ΔrocG) led to a dramatic decrease in production of the alkaline cellulase Egl-237, even in spite of an observed increase in cell yield [9]. At that time, it remained unclear if this phenomenon is caused by acidification of the growth medium, as in the case of alkaline α-amylase production. Here, we investigated the mechanisms underlying decreased enzyme production in strain 874ΔrocG and attempted to boost production of alkaline cellulase Egl-237 by overcoming the rate-limiting factors we identified.

## Results and discussion

### Growth characteristics of strains MGB874 and 874ΔrocG producing alkaline cellulase Egl-237

In our recent study, we found that deletion of the *rocG* gene in the genome-reduced strain MGB874 dramatically decreased the level of production of the alkaline cellulase Egl-237, despite an increase in cell yield [9]. Previous studies showed that mutations in *rocG* result in the rapid accumulation of suppressor mutations in *gudB*, a second, cryptic glutamate dehydrogenase gene harboring an insertion of three amino acids with respect to the common ancestral GluDH sequence [11,19,20]. However, sequence analysis of *gudB* alleles in strains MGB874 and 874ΔrocG revealed that the insertion mutation of the three amino acids has been retained in these strains.

**Figure 2:**
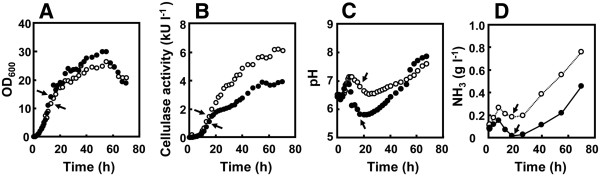
**Growth characteristics of strains MGB874 and 874**Δ**rocG producing the alkaline cellulase Egl-237.** Strains MGB874 (open circles) or MGB874∆rocG (closed circles) were transformed with pHYS237 for production of alkaline cellulase Egl-237. The strains were cultured in 2xL medium containing 7.5% (w/v) maltose monohydrate by batch fermentation with a 30-L jar fermentor. Cell yield (**A**), extracellular cellulase activity (**B**), external pH of the growth media (**C**) and ammonia concentration in the growth media (**D**) were measured at the indicated times. Arrows indicate the point at which transcriptome analyses were conducted.

To obtain insight into the mechanism responsible for decreased enzyme production in strain 874ΔrocG, we conducted time course analyses of production of alkaline cellulase Egl-237 in strains MGB874 and 874ΔrocG under batch fermentation conditions achieved using a 30-liter jar fermentor. As shown in Figure 2A and 2B, after the transition phase, production of alkaline cellulase Egl-237 in strain 874ΔrocG dramatically decreased as compared with strain MGB874, although the cell yield in strain 874ΔrocG was higher. Additionally, in the culture medium at the transition phase, we observed a decrease in pH and ammonium depletion for strain 874ΔrocG as compared to strain MGB874 during cultivation (Figure 2C and 2D).

### Comparison of transcriptome profiles of strains MGB874 and 874ΔrocG

**Figure 3:**
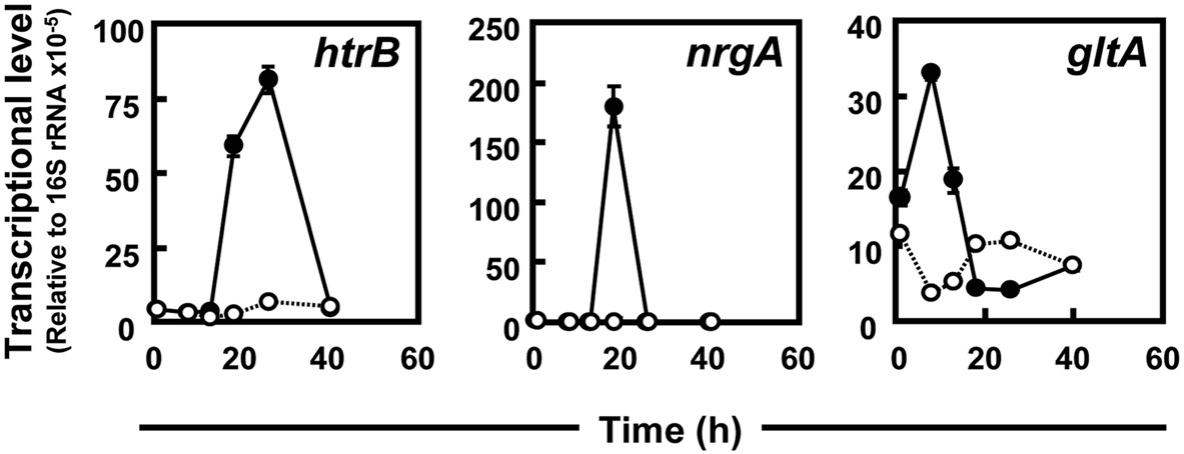
**Time course of transcription during growth of *B. subtilis *strains MGB874 and 874**Δ**rocG.** The strains MGB874 (open circles) and 874∆rocG (closed circles) were transformed with pHYS237. The transformants were cultured using shake-flask fermentation. Transcript levels for *htrB, nrgA* and *gltA* were determined by qRT-PCR (primers shown in Additional file 2: Table S1). Transcript levels were normalized to 16S rRNA levels. Error bars represent standard deviations (*n*=3).

We then compared transcriptome profiles of MGB874 and 874ΔrocG cells at transition phase (at 18 h, indicated by arrow in Figure 2) using a custom Affymetrix tilling chips. The top-ranked 20 up-regulated genes and bottom-ranked 20 down-regulated genes in 874ΔrocG cells were listed in Tables 1 and 2, respectively. Firstly, we found that expression of *htrA* was markedly induced in 874ΔrocG cells (Table 1). Our previous study revealed that the decrease in external pH impaired secretion of alkaline α-amylase AmyK38 in strain MGB874, and induced *htrA* and *htrB* expression [16]. Indeed, expression of *htrB* was also induced in 874ΔrocG cells (4.62 fold) as compared to strain MGB874 cells in our transcriptome analysis. Additionally, time course analysis using qRT-PCR confirm that *htrB* expression is up-regulated in 874ΔrocG cells during early stationary phase (from 18 to 24 h, Figure 3). These results suggest that acidification of the growth medium might impair secretion of alkaline cellulase Egl-237 in 874ΔrocG cells.

**Table 1:** **Genes up-regulated in 874**Δ**rocG cells (top-ranked 20 genes)**

**Gene**^**a**^	**Product**^**a**^	**Function**^**a**^	**Average signal**^**b**^		**Ratio**^**c**^	**Transcriptional factor**^**d**^
			**MGB874**	**874**Δ**rocG**		
*nrgA*	ammonium transporter	ammonium uptake	208	3926	18.91	**TnrA(+)**
*yvrI*	co-sigma factor with YvrHa	RNA polymerase sigma factor	92	1266	13.78	YvrH(+)
*ykzB*	unknown	unknown	92	1078	11.72	**TnrA(+)**
*nasC*	nitrate reductase (catalytic subunit)	utilization of nitrate	128	1463	11.46	GlnR(−), **TnrA(+)**
*ansZ*	asparaginase	asparagine utilization	202	1976	9.78	**TnrA(+)**
*tnrA*	transcription activator/ repressor	regulation of nitrogen assimilation	103	981	9.56	**TnrA(+)**
*nasA*	nitrate transporter	nitrate uptake	227	1975	8.7	GlnR(−), **TnrA(+)**
*yvmB*	unknown	unknown	119	977	8.23	
*yjgD*	unknown	survival of ethanol stress	158	1185	7.51	
*nasB*	nitrate reductase (electron transfer subunit)	utilization of nitrate	180	1346	7.49	GlnR(−), **TnrA(+)**
*htrA*	serine protease Do	protein quality control	719	4742	6.6	CssR(+), HtrA(−)
*ygxB*	unknown	unknown	184	1156	6.29	
*yqzH*	unknown	unknown	248	1463	5.9	LexA(−)
*spoVFB*	dipicolinate synthase (subunit B)	dipicolic acid production	82	475	5.8	
*nrgB*	nitrogen-regulated PII-like protein	regulation of ammonium uptake	821	4727	5.76	**TnrA(+)**
*ntdA*	sugar aminotransferase	synthesis of antibiotic neotrehalosadiamine	133	707	5.33	YhjM(+)
*bmrU*	multidrug resistance protein	multidrug resistance	180	949	5.28	
*yrbD*	sodium/proton-dependent alanine transporter	uptake of alanine	327	1721	5.27	
*yitT*	unknown	unknown	247	1301	5.26	
*yuzA*	unknown	unknown	146	758	5.2	

^a^The SubtiWiki was used as a reference for the genes, products and functions [21].

^b^The average signal intensities of probes in each coding sequence.

^c^The ratio of each of the genes was obtained by dividing the average signal intensity in each coding sequence of 874ΔrocG cells by that for MGB874 cells.

^d^The Database of Transcriptional Regulation in *Bacillus subtilis* (DBTBS) was used as a reference [22]. Transcriptional activators or repressors are indicated by a (+) or (−), respectively. TnrA is shown in bold type.

**Table 2:** **Genes down-regulated in 874**Δ**rocG cells (bottom-ranked 20 genes)**

**Gene**^**a**^	**Product**^**a**^	**Function**^**a**^	**Average signal**^**b**^		**Ratio**^**c**^	**Transcriptional factor**^**d**^
			**MGB874**	**874ΔrocG**		
*yuiA*	unknown	unknown	4307	533	0.12	
*yycC*	unknown	unknown	1915	273	0.14	**TnrA(−)**
*yycB*	unknown	unknown	1883	287	0.15	**TnrA(−)**
*dhbC*	isochorismate synthase	biosynthesis of the siderophore bacillibactin	2560	529	0.21	Fur(−)
*pel*	pectate lyase C	degradation of polygalacturonic acid	3736	808	0.22	ComA(+), **TnrA(−)**
*ilvB*	acetolactate synthase (large subunit)	biosynthesis of branched-chain amino acids	2106	459	0.22	CcpA(+), CodY(−), **TnrA(−)**, TrnS-Leu2(+)
*leuB*	3-isopropylmalate dehydrogenase	biosynthesis of leucine	2072	467	0.23	CcpA(+), CodY(−), **TnrA(−)**, TrnS-Leu2(+)
*dhbF*	unknown	biosynthesis of the siderophore bacillibactin	2081	474	0.23	Fur(−)
*serA*	phosphoglycerate dehydrogenase	biosynthesis of serine	2575	603	0.23	
*leuA*	2-isopropylmalate synthase	biosynthesis of leucine	1680	396	0.24	CcpA(+), CodY(−), **TnrA(−)**, TrnS-Leu2(+)
*dhbB*	isochorismatase	biosynthesis of the siderophore bacillibactin	1897	447	0.24	Fur(−)
*leuC*	3-isopropylmalate dehydratase (large subunit)	biosynthesis of leucine	2166	516	0.24	CcpA(+), CodY(−), **TnrA(−)**, TrnS-Leu2(+)
*ilvC*	ketol-acid reductoisomerase (2,3-dihydroxy-3-methylbutanoate, 2-acetolactate)	biosynthesis of branched-chain amino acids	2981	713	0.24	CcpA(+), CodY(−), **TnrA(−)**, TrnS-Leu2(+)
*yocS*	putative sodium-dependent transporter	unknown	1028	248	0.24	
*yodF*	unknown	unknown	737	182	0.25	**TnrA(−)**
*ydzA*	unknown	unknown	1035	266	0.26	
*yxxG*	unknown	unknown	435	119	0.27	DegU(−), YvrH(+)
*dhbE*	2,3-dihydroxybenzoate-AMP ligase (enterobactin synthetase component E	biosynthesis of the siderophore bacillibactin	1539	439	0.29	Fur(−)
*leuD*	3-isopropylmalate dehydratase (small subunit)	biosynthesis of leucine	1137	327	0.29	CcpA(+), CodY(−), **TnrA(−)**, TrnS-Leu2(+)
*yuiB*	unknown	unknown	3785	1150	0.3	

^a^The SubtiWiki was used as a reference for the genes, products and functions [21].

^b^The average signal intensities of probes in each coding sequence.

^c^The ratio of each of the genes was obtained by dividing the average signal intensity in each coding sequence of 874ΔrocG cells by that for MGB874 cells.

^d^The Database of Transcriptional Regulation in *Bacillus subtilis* (DBTBS) was used as a reference [22]. Transcriptional activators or repressors are indicated by a (+) or (−), respectively. TnrA is shown in bold type.

Importantly, we found that many of the genes that are activated or repressed in 874ΔrocG cells are controlled by the transcriptional factor TnrA. Indeed, 10 of the bottom-ranked 20 genes and 8 of the top-ranked 20 genes were members of the TnrA regulon (Tables 1 and 2). TnrA is a major transcription factor in *B. subtili*s that controls gene expression under nitrogen-limited growth [23-25]. Time course analysis revealed that *nrgA,* an ammonia transporter gene regulated by TnrA, is transiently up-regulated in 874ΔrocG cells just before cells enter the stationary phase (at 18 h, Figure 3), which corresponds to the point that ammonium depletion occurs in the culture medium during culture of strain 874ΔrocG (Figure 2D). These results clearly indicate that nitrogen starvation is induced in 874ΔrocG cells likely due to ammonium depletion in the culture medium.

Expression of glutamate synthase (GltAB) is also known to be negatively regulated by TnrA [26], in addition to its regulation by GltC. Indeed, expression of *gltA* in 874ΔrocG cells significantly decreased after depletion of ammonium to levels lower than that in MGB874 cells, although *gltA* levels in 874ΔrocG cells were much higher than levels in MGB874 cells before entering stationary phase (at 18 h, Figure 3). These results indicate that although activation of the glutamate synthetic pathway is induced via deletion of *rocG* during the early growth phase as expected, it is subsequently suppressed by depletion of ammonium in the culture medium.

### Cultivation using the NH_3_-pH auxostat approach improves enzyme production in strain 874ΔrocG

**Figure 4:**
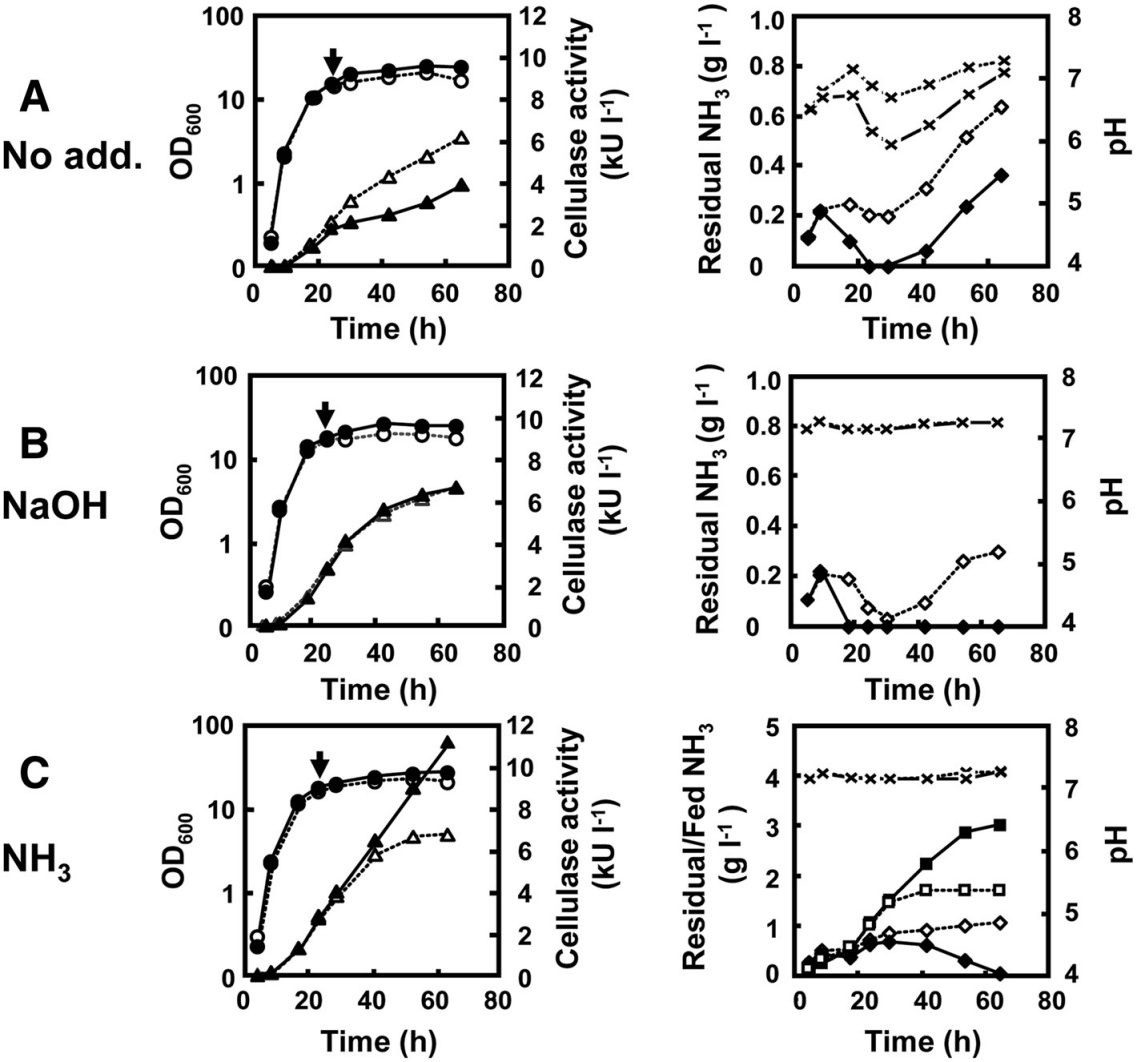
**Growth characteristics of strains MGB874 and 874**Δ**rocG producing alkaline cellulase Egl-237.** The strains MGB874 (open symbols and dotted lines) and 874∆rocG (closed symbols and solid lines) were transformed with pHYS237, and cultured in 2xL medium containing 12.5% (w/v) maltose monohydrate by the pH-Stat fermentation using 2-L jar fermentor. Fermentation without pH control was done as a reference (**A**). The pH was adjusted to 7.2 by the automatic addition of 1M NaOH (**B**) or 10% (w/v) aqueous NH_3_ (**C**). Cell yields (circles) and extracellular cellulase activities (triangles) were shown on the left side of the figure. Residual ammonia concentrations (diamonds) and external pHs (crosses) in the growth media were shown on the right side of the figure. Additionally, total amounts of ammonia fed (squares) were also displayed under the NH_3_-pH auxostat. Arrows indicate the point at which transcriptional analysis was conducted.

To exclude the influence of decreased pH and depletion of ammonia in the growth medium associated with culture of strain 874ΔrocG, we next performed pH-stat fermentation using NaOH or aqueous NH_3_ and a 2-L jar fermentor (Figure 4). The pH of the growth media was adjusted to 7.2, which corresponds to the highest pH observed in the growth medium of strain MGB874 in the absence of pH control (Figure 4A). Additionally, to prevent the carbon source from becoming a limiting factor, the initial concentration of maltose in the growth media was increased from 7.5% to 12.5%, which is sufficient in these fermentation conditions (data not shown).

When fermentation was performed without pH control, the growth characteristics were similar to the results shown in Figure 2. On the other hand, when pH-stat fermentation using NaOH was performed, the production of alkaline cellulase Egl-237 in strain 874ΔrocG was improved to nearly the same level as that observed for strain MGB874 (Figure 4B). In both these cases, the concentrations of ammonia were significantly decreased as compared to those reached during cultivation without pH control. To examine if the decrease in ammonia affects production of alkaline cellulase Egl-237, we performed pH-stat fermentation using aqueous NH_3_, using a so-called NH_3_-pH auxostat [27] (Figure 4C). The enzyme production period in strain 874ΔrocG was prolonged with use of the NH_3_-pH auxostat, whereas the production profile of alkaline cellulase Egl-237 in MGB874 cells was similar in both cultivation conditions. With the NH_3_-pH auxostat, the production of alkaline cellulase Egl-237 in strain 874ΔrocG was 1.67-fold higher than that in strain MGB874 at the end of the cultivation period (Figure 4C). Production of alkaline cellulase Egl-237 in strain 874ΔrocG corresponded to about 5.5 g l^-1^, the highest level reported so far [6].

Notably, the level of residual ammonia in the growth medium from strain 874ΔrocG was lower than that from strain MGB874, although the total amount of ammonia introduced into the growth medium for strain 874ΔrocG was considerably larger than that for strain MGB874 (Figure 4C). These data suggest that the ratio of assimilated ammonia in 874ΔrocG cells was higher than that in MGB874 cells and furthermore, that assimilation activity is maintained through late stages of cultivation.

It should be noted that the *rocG* deletion in wild-type strain 168 (168ΔrocG) also enhanced production of alkaline cellulase Egl-237 with the NH_3_-pH auxostat (Additional file 1: figure S1). In this experiment, we also confirmed that the insertion mutation of the three amino acids was retained in strain 168ΔrocG. Notably, the production level from strain 168ΔrocG (2.8 g l^-1^) was about half of that of strain 874ΔrocG, indicating the importance of the genetic background of the reduced-genome strain for higher levels of alkaline cellulase Egl-237 production.

### Changes in gene expression underlying improvement in enzyme production with pH-stat fermentation

**Figure 5:**
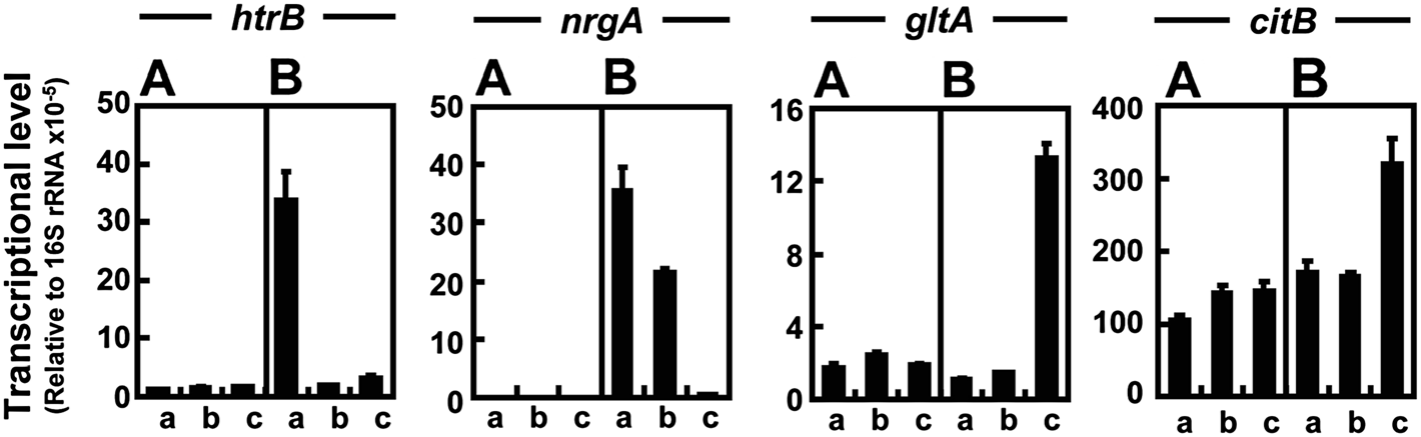
**Transcriptional levels of selected genes in strains MGB874 and 874**Δ**rocG.** The strains MGB874 (**A**) and 874∆rocG (**B**) were transformed with pHYS237, and cultured by the pH-Stat fermentation. Fermentation without pH control was done as a reference (**a**). The pH was adjusted to 7.2 by addition of NaOH (**b**) or aqueous NH_3_ (**c**). RNA was isolated from the cells at the 24 h of the cultivation time (indicated by arrows in Figure 4), and expression level of *htrB, nrgA*, *gltA* and *citB* were determined by qRT-PCR. The transcriptional levels are expressed relative to those of 16S rRNA. Error bars represent standard deviations (*n*=3).

To investigate the changes in gene expression underlying the improvement of enzyme production in 874ΔrocG cells under pH-stat fermentation, RNA was extracted from cells at 24 h of cultivation (Figure 4; arrows), and expression levels of selected genes were measured by qRT-PCR (Figure 5). Although transcriptional levels for selected genes were not significantly changed in MGB874 cells under any fermentation conditions, remarkable changes in expression of these genes were observed in 874ΔrocG.

Firstly, expression of *htrB* was reduced to the same level in strain MGB874 under fermentation conditions with pH-control not only with aqueous NH_3_ but also with NaOH, clearly indicating that the CssRS-dependent secretion stress response is induced by overproduction of alkaline cellulase Egl-237 in 874ΔrocG cells under the low external pH condition (Figure 5). Notably, production of alkaline cellulase Egl-237 did not induce the secretion stress response in MGB874 cells (Figures 3 and 5) but overproduction of alkaline α-amylase AmyK38 induced this response to a high degree in MGB874 cells [16]. Because the decrease of external pH was more severe in 874ΔrocG cultivation (without pH control) compared to that in MGB874 cultivation (Figure 4A), the threshold value of external pH leading to secretion stress responses for overproduction of alkaline cellulase Egl-237 would be lower than for the overproduction of AmyK38.

We also found that expression of *nrgA,* known to be activated under nitrogen-limited growth*,* was down-regulated in 874ΔrocG cells using the NH_3_-pH auxostat, suggesting avoidance of nitrogen starvation (Figure 5). Furthermore, the expression level of *gltA*, encoding a subunit of glutamate synthase, was 9-fold higher in 874ΔrocG cells than in MGB874 when using the NH_3_-pH auxostat (Figure 5). As ammonia can be assimilated via the glutamine synthetase-glutamate synthase (GS-GOGAT) pathway in *B. subtilis* (Figure 1), activation of the glutamate synthetic pathway might indirectly contribute to the enhancement of ammonia assimilation ability in 874ΔrocG cells (Figure 4C and 5). With the NH_3_-pH auxostat, the continuous conversion of ammonia to glutamate in 874ΔrocG cells might lead to increased flux in the synthesis of other amino acids via transamination, resulting in enhanced production of alkaline cellulase Egl-237.

As mentioned, enzyme productivity lasted through the end of the cultivation period in strain 874ΔrocG with use of the NH_3_-pH auxostat but this was not observed for strain MGB874 under the same conditions (Figure 4C). Activation of the glutamate synthetic pathway in 874ΔrocG cells could account for this difference. Furthermore, we found that expression of the gene encoding aconitase (*citB*) was up-regulated in 874ΔrocG cells but not MGB874 cells under NH_3_-pH auxostat (Figure 5). Expression of *citB* has been reported to be indirectly repressed by 2-oxoglutarate, which competitively represses the reaction of citrate synthase (CitZ), leading to repression of *citB* by the transcriptional regulator CcpC in the absence of the effector citrate [28,29]. Therefore, improvement of metabolic flux from 2-oxoglutarate to glutamate in strain 874ΔrocG might lead to activation of *citB* due to inactivation of the repressor CcpC*.* Blencke *et al*. reported that TnrA exerts a weak activating effect on *citB* expression [30]. However, in our experiment, the expression levels of TnrA regulated gene *nrgA* were almost the same in strains MGB874 and 874ΔrocG under NH_3_-pH auxostat (Figure 5). Thus, it seems that TnrA did not participate in activation of *citB* in strain 874ΔrocG, compared to that of strain MGB874 under NH_3_-pH auxostat. Activation of *citB* might contribute to prolonged high enzyme productivity through the generation of reducing power via the tricarboxylic acid (TCA) cycle.

## Conclusion

Here, we describe conditions resulting in the highest levels of production of alkaline cellulase Egl-237 in *B. subtilis* cells reported to date. We found that deletion of the glutamate dehydrogenase gene *rocG* in the genome-reduced strain MGB874 (874ΔrocG) and cultivation of 874ΔrocG using NH_3_-pH auxostat conditions leads to enhanced enzyme production through prolonged high enzyme productivity until the end of cultivation. This beneficial effect is very likely a consequence of an enhanced metabolic flux from 2-oxoglutarate to glutamate and generation of metabolic energy through activation of the TCA cycle.

Additionally, we found that the overproduction of alkaline cellulase Egl-237 causes the induction of CssRS-dependent secretion stress responses in the acidified growth medium below the threshold pH value, which is lower than that for the overproduction of alkaline α-amylase AmyK38.

With the NH_3_-pH auxostat, levels of alkaline cellulase Egl-237 produced by strain 874ΔrocG far exceeded those produced by the wild-type genetic-background strain 168ΔrocG, and reached the highest level reported so far, corresponding to 5.5 g/L. However, it is not clear at the moment if these improvements are attributable to a global synergistic effect of large-scale genome reduction or to individual effects of one or more specific gene deletions. To further improve enzyme production, we are presently attempting to elucidate the mechanisms underlying the improvement in productivity we have observed.

## Materials and methods

### Bacterial strains, plasmids, and growth media

The bacterial strains and plasmids used in this study are listed in Table 3. *E. coli* HB101 (Takara Bio Inc.) was used as the host for plasmid preparation and was grown in Luria-Bertani (LB) medium [1% (w/v) Bacto tryptone (Difco), 0.5% (w/v) Bacto yeast extract (Difco), and 1% (w/v) NaCl]. Strain 168ΔrocG, a *rocG* mutant strain derived from strain 168, was constructed in a similar way to construction of strain 874ΔrocG, which was described previously [9]. *B. subtilis* mutant strains were transformed with the plasmid pHYS237 for production of alkaline cellulase Egl-237, which originated from *Bacillus* sp. strain KSM-S237 [9], using the protoplast transformation method [31]. For enzyme production, we used 2xL medium [2% (w/v) Bacto tryptone, 1% (w/v) Bacto yeast extract, 1% (w/v) NaCl, 7.5 μg ml^-1^ manganese sulfate 4–5 hydrate, and 15 μg ml^-1^ Tet] supplemented with 7.5% (w/v) or 12.5% (w/v) maltose monohydrate.

**Table 3:** Bacterial strains and plasmids used in or constructed for this study

**Strain or plasmid**	**Relevant properties**^**†**^	**Source or reference**
Strain		
*Bacillus subtilis*		
168	*trpC2*	[4]
168∆rocG	*trpC2* ∆*rocG*::*spec*	This study
MGB874	*trpC2* ∆prophage1-6 ∆PBSX ∆SPβ ∆*pks* ∆skin ∆*pps*∆ (*ydeK-ydhU*) ∆(*yisB-yitD*) ∆(*yunA-yurT*) ∆(*cgeE-yodU*) ∆(*ypqP-ypmQ*) ∆(*yeeK-yesX*)∆(*pdp-rocR*) ∆(*ycxB-sipU*) ∆(*yrkS-yraK*) ∆(*sboA-ywhH*) ∆(*yyb-yyaJ*) ∆(*yncM-yndN*)	[6]
874∆rocG	MGB874 ∆*rocG*::*spec*	[9]
*Escherichia coli*		
HB101	*supE44* ∆*(mcrC-mrr) recA13 ara-14 proA2 lacY1 galK2 rpsL20 xyl-5 mtl-1 leuB6 thi-1*	Takara Bio
Plasmid		
pHY300PLK	Shuttle vector for *E. coli* and *B. subtilis*	Takara Bio
pHYS237	pHY300PLK carrying the gene for alkaline endo-1,4-β-glucanase (Egl-237) from *Bacillus* sp. strain KSM-S237, containing *amp* and *tet*	[6]

^†^Antibiotic resistance genes are abbreviated as follows: *amp*, ampicillin; *tet*, tetracycline; *spec*, spectinomycin; *neo*, neomycin.

### Culture methods for the assessment of alkaline cellulase Egl-237 production

For shake-flask fermentation, transformants were pre-cultured in LB medium with 15 μg ml^-1^ Tet with shaking at 120 rpm at 30°C for 15 h, and 600 μl of the pre-culture was inoculated into 30 ml of 2×L medium with 7.5% (w/v) maltose monohydrate in a 500-ml Sakaguchi flask.

For jar fermentation, *B. subtilis* harboring pHYS237 stored in 10% glycerol at −80°C were inoculated onto LB agar medium with 15 μg ml^-1^ Tet. After incubation at 37°C for 12 h, cells were collected and inoculated into pre-culture medium at an optical density at 600 nm (OD_600_) of 0.02. For batch fermentation, cells were pre-cultured in 200 ml of 2×L medium with 7.5% (w/v) maltose monohydrate with shaking at 210 rpm at 30°C to an OD_600_ of 0.3 to 0.5, then inoculated into a 30-L jar fermentor (working volume, 18 liters). The 30-L jar fermentor was operated at an aeration rate of 0.4 vvm and an agitation rate of 300 rpm. For pH-stat fermentation, cells were pre-cultured in 30 ml of 2×L medium with 12.5% (w/v) maltose monohydrate with shaking at 120 rpm at 30°C to an OD_600_ of 0.3 to 0.5, then inoculated into a 2-L jar fermentor (working volume, 0.8 liters). The 2-L jar fermentor was operated at an aeration rate of 0.5 vvm and an agitation rate of 800 rpm. The pH was kept at 7.2 via automatic addition of 1M NaOH or 10% (w/v) aqueous NH_3_. Fermentation without pH adjustments was used as a control. As appropriate to specific assays, cultured cells were removed by centrifugation at 9,000 × *g* and the supernatants were stored at −30°C. For RNA extraction, cells were separated by centrifugation, washed with 10 mM Tris–HCl (pH 7.5), frozen with liquid nitrogen, and stored at −80°C.

### Analytical methods and cellulase activity

The concentration of ammonia and maltose in the culture supernatants was determined by enzymatic analysis according to the F-Kit UV method (Boehringer GmbH). Cellulase activity in the culture medium was determined as described previously [9]. The amount of enzyme required for release of 1 μmol of *p-*nitrophenol per minute was defined as 1 U.

### High-resolution transcriptome analysis

Total RNA was extracted from *B. subtilis* cells as described previously [32]. Synthesis of cDNA, terminal labeling, and oligonucleotide chip hybridization were performed as described in the Affymetrix instruction manual. Transcriptional signals were analyzed and visualized along genome coordinates using the program IMC Array Edition (In Silico Biology, Japan). The signal intensities of each experiment were adjusted to confer a signal average of 500 and normalized by MA plot analysis for comparison of strains MGB874 and 874ΔrocG. [33,34]. The average signal intensities of probes in each coding sequence were calculated after removal of the lowest and highest intensities.

### Quantitative real-time PCR

Quantitative real-time PCR (qRT-PCR) amplification, detection, and analysis were performed with the Mx3005P Real-time PCR system (Stratagene) and Brilliant II Fast SYBR Green QPCR Master Mix (Stratagene), as previously described [9]. The sequences of the primers used in real-time PCR were developed with Primer 3 (version 0.4.0) [35] and are listed in Additional file 2: Table S1 in the supplemental material. Experimental RNA levels were normalized to 16S rRNA levels, as previously described [16].

## Abbreviations

GRAS: generally regarded as safe; CssRS: Control secretion stress Regulator and Sensor; qRT-PCR: quantitative real-time PCR; NaCl: sodium chloride; Tet: tetracycline.

## Competing interests

The content of this manuscript is relevant to a patent application made by Kao Corporation (Patent no. JP2007-330255A); however, all authors declare that they have no competing interests.

## Authors’ contributions

NO, KO, and KA initiated and coordinated the project. KM and YK drafted the manuscript, constructed mutant strains, evaluated production levels of alkaline cellulase Egl-237, and measured pH and ammonium concentrations in the growth medium. KM and TM performed tilling array and qPCR. ES supported our results by metabolic analysis. HT and SK processed tilling array data. NO and KO supervised the study and reviewed results. All authors have read and approved the final manuscript.

## Supplementary Material

Additional file 1: Figure S1 Cell yield and alkaline cellulase Egl-237 production under the NH_3_-pH auxostat, The alkaline cellulase Egl-237 overproducing strains in the presence (+) or absence (−) of *rocG* were cultured by the pH-Stat fermentation. The pH was adjusted to 7.2 by addition aqueous NH_3_. The cell yields (at 42 h; open circles) and the cellulase activities in growth media (72h; black bars) were measured. (A) The wild-type strain 168 and strain 168∆rocG. (B) The genome-reduced strain MGB874 and strain 874∆rocG. Click here for file

Additional file 2: Table S1 Oligonucleotide primers used for real-time PCR analysis. Click here for file
