# Supplementary figures and images for: Improved production of secreted heterologous enzyme in Bacillus subtilis strain MGB874 via modification of glutamate metabolism and growth conditions
Source: Microb Cell Fact. 2013 Feb 18;12:18. doi: 10.1186/1475-2859-12-18 (PMC3600796; doi:10.1186/1475-2859-12-18)

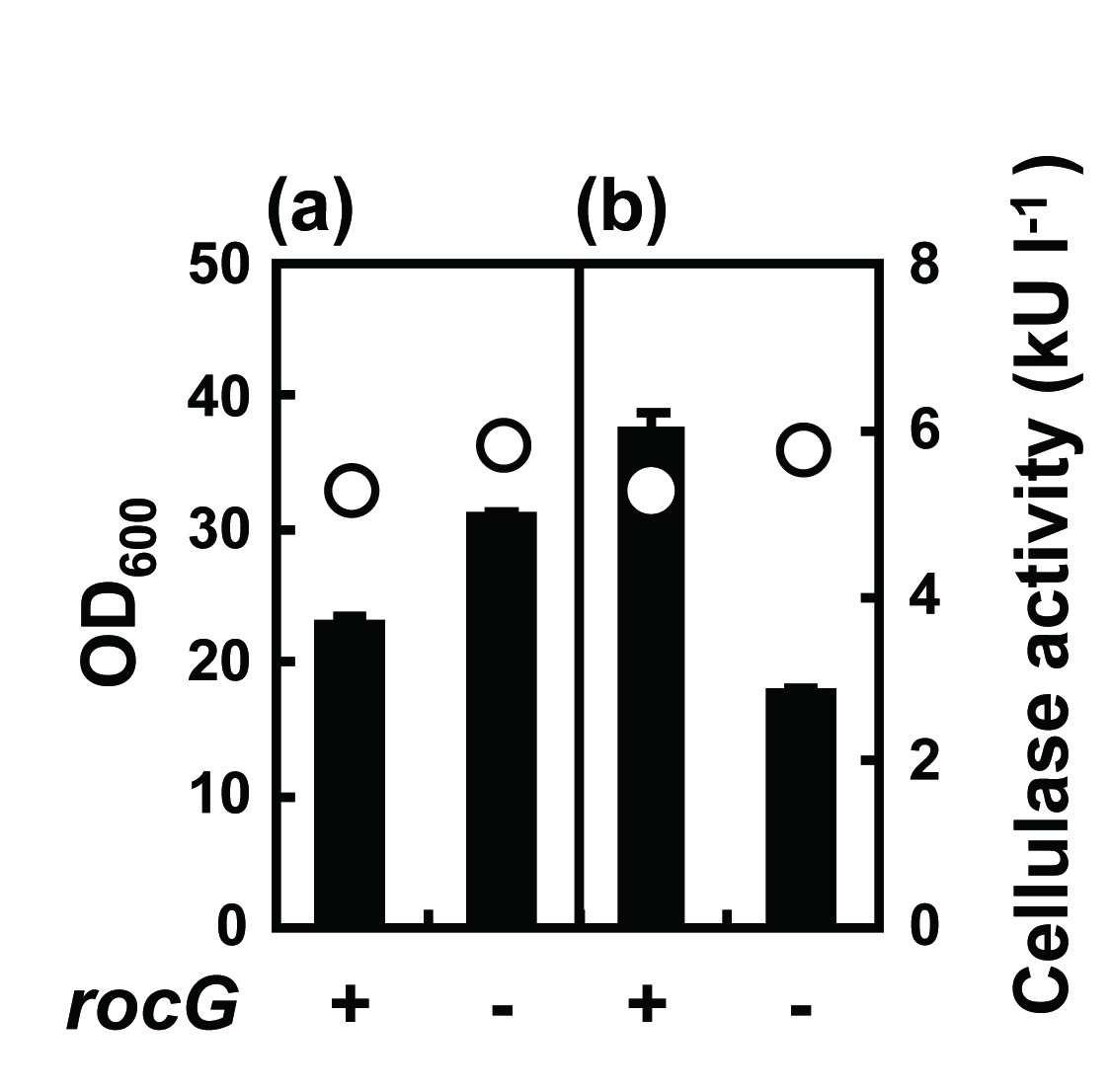

Supplement: Additional file 1: Figure S1 — Cell yield and alkaline cellulase Egl-237 production under the NH3-pH auxostat, The alkaline cellulase Egl-237 overproducing strains in the presence (+) or absence (−) of rocG were cultured by the pH-Stat fermentation. The pH was adjusted to 7.2 by addition aqueous NH3. The cell yields (at 42 h; open circles) and the cellulase activities in growth media (72h; black bars) were measured. (A) The wild-type strain 168 and strain 168∆rocG. (B) The genome-reduced strain MGB874 and strain 874∆rocG. [file 1475-2859-12-18-S1.jpeg]
