# Supplementary material for: Improved production of secreted heterologous enzyme in Bacillus subtilis strain MGB874 via modification of glutamate metabolism and growth conditions
Source: Microb Cell Fact. 2013 Feb 18;12:18. doi: 10.1186/1475-2859-12-18 (PMC3600796; doi:10.1186/1475-2859-12-18)
Supplement: Additional file 2: Table S1 — Oligonucleotide primers used for real-time PCR analysis. [file 1475-2859-12-18-S2.doc]

**Additional file 2: Table S1** Oligonucleotide primers used for real-time PCR analysis

| Target gene | Forward primer (5'-3') | Reverse primer (5'-3') |
| --- | --- | --- |
| 16S rRNA | TCCGCAATGGACGAAAGTCT | ACGATCCGAAAACCTTCATCA |
| *htrB* | ACCGGACAATTGATGTGGAT | CACCTTTGCCCAATTGATCT |
| gltA | GGGCGTCGTAAAAGTGATGT | CTCCAACTTCAAAGGCTTGC |
| nrgA | TTTTGCGCTTTACTCGTGTG | ACCCCGGAGGAAATATGAAC |
| *citB* | GACGTATTCACCCGCTTGTT | GTGACTGAATCGCCGAATTT |
